# *Mimosa* *pudica* L. aqueous extract protects mice against pilocarpine–picrotoxin kindling-induced temporal lobe epilepsy, oxidative stress, and alteration in GABAergic/cholinergic pathways and BDNF expression

**DOI:** 10.3389/fphar.2024.1301002

**Published:** 2025-02-10

**Authors:** Hart Mann Alain Youbi Mambou, Simon Pale, Orelien Sylvain Mtopi Bopda, Vanessa Tita Jugha, Nji Seraphin Ombel Musa, Tambong Ako Ojongnkpot, Bertrand Yuwong Wanyu, Raymond Bess Bila, Rashed N. Herqash, Abdelaaty A. Shahat, Germain Sotoing Taiwe

**Affiliations:** ^1^ Department of Animal Biology and Conservation, Faculty of Science, University of Buea, Buea, Cameroon; ^2^ Department of Pharmacognosy, College of Pharmacy, King Saud University, Riyadh, Saudi Arabia

**Keywords:** *Mimosa pudica*, temporal lobe epilepsy, oxidative stress, GABAergic status, cholinergic status, brain-derived neurotrophic factors

## Abstract

Ethnopharmacological studies revealed that the leaves and stems of *Mimosa pudica* L. (Fabaceae) are widely used for the treatment of epilepsy. This study sought to investigate the effects of the aqueous extract of *Mimosa pudica* leaves and stems against pilocarpine–picrotoxin kindling-induced temporal lobe epilepsy in mice and its implication on oxidative/nitrosative stress, GABAergic/cholinergic signalling, and brain-derived neurotrophic factor (BDNF) expression. The animals were treated for seven consecutive days as follows: one normal group and one negative control group that received orally distilled water; four test groups that received orally four doses of *Mimosa pudica* (20, 40, 80, and 160 mg/kg), respectively; and one positive control group that received 300 mg/kg sodium valproate intraperitoneally. One hour after the first treatment (first day), *status epilepticus* was induced by intraperitoneal injection of a single dose of pilocarpine (360 mg/kg). Then, 23 hours after the injection of pilocarpine to the mice, once again, they received their different treatments. Sixty minutes later, they were injected with a sub-convulsive dose of picrotoxin (1 mg/kg), and the anticonvulsant property of the extract was determined. On day 7, open-field, rotarod, and catalepsy tests were performed. Finally, the mice were sacrificed, and the hippocampi were isolated to quantify some biochemical markers of oxidative/nitrosative stress, GABAergic/cholinergic signalling, and BDNF levels in the hippocampus. *Mimosa pudica* extracts (160 mg/kg) significantly increased the latency time to *status epilepticus* by 70.91%. It significantly decreased the number of clonic and tonic seizures to 9.33 ± 1.03 and 5.00 ± 0.89, and their duration to 11.50 ± 2.07 and 6.83 ± 0.75 s, respectively. Exploratory behaviour, motor coordination, and catalepsy were significantly ameliorated, respectively, in the open-field, rotarod, and catalepsy tests. Pilocarpine–picrotoxin-induced alteration of oxidant–antioxidant balance, GABA-transaminase stability, acetylcholinesterase/butyrylcholinesterase activity, and neurogenesis were attenuated by the extract (80–160 mg/kg). This study showed that the aqueous extract of *Mimosa pudica* leaves and stems ameliorated epileptogenesis of temporal lobe epilepsy and could be used for the treatment of temporal lobe epilepsy.

## 1 Introduction

Epileptic patients witness chronic neurological disorder characterized by recurrent unprovoked and spontaneous seizures, which is triggered by an abnormal electrical phenomenon in the brain (Kanner and Balabanov, 2002). Globally, an estimated 70 million individuals are affected by epilepsy (Tang et al., 2017), with sub-Saharan Africa (0.9%) and central Africa (6.0%) documenting the highest prevalence rates of epilepsy in Africa (Ba-Diop et al., 2014). The rate in Cameroon is 25‒66/1,000 and might be the highest in the world, with a typical prevalence of 6.6% (Boullé et al., 2019).

During epileptic convulsions, the activation of voltage-gated glutamate receptors causes a significant augmentation in the level of Ca^2+^ and Na^+^ influxes, accompanied by the transport of Cl^−^ and H_2_O across the neuronal membranes. Activation of glutamate receptors followed by excitotoxicity, including suppression of axon sprouting by inhibitory GABAergic interneurons, can generate an epiphenomenon related to spontaneous seizures (Kanaani et al., 2010; Morimoto et al., 2004). These processes lead to epileptogenesis causing development of oxidative/nitrosative stress, neuroinflammation, and brain-derived neurotropic factor (BDNF) expression (Grosso and Geronzi, 2014; Ignacio-Mejía et al., 2023; Wang et al., 2021).

Due to the complexity of epileptogenesis and epilepsy physiopathologies taken individually and the substitute of mechanisms implicated when these pathologies are progressing, their management is more than challenging. During the last few decades, increasing efforts have been made to overcome this complex syndrome. Antiepileptic drugs act by reducing the excitability of neuronal membranes by interacting with a number of neuromediators, receptors, and ion channels (Rossetti and Lowenstein, 2011). Unfortunately, drug management of temporal lobe epilepsy faces a significant therapeutic challenge despite the substantial progress made (Kwan et al., 2010; Simonato et al., 2014). Current antiepileptic drugs used against temporal lobe epilepsy neither provide a cure nor prevent relapse. In addition, antiepileptic drugs can neither stop neurodegeneration nor reverse the apoptosis and necrosis of neurons (Perucca and Gilliam, 2012). Hence, the development of a newer, safer, more effective and affordable pharmacological agent has become a major goal in epilepsy research.

Drugs/medicine stemmed from plants are a good alternative as they constitute a patent source of new metabolites, which are important to antagonise epileptogenesis, break the progression of epileptic seizures, and optimize therapeutic efficacy, with fewer side effects. The plant kingdom is a major target in the search for new drugs and lead compounds. In addition, herbal medicine remains the hope of about 80% of the population worldwide, mainly in developing countries (Taiwe et al., 2015; Taiwe et al., 2016a). Ethnopharmacological studies have revealed that the *Mimosa pudica* L. (Fabaceae) extract is used in traditional medicine for the treatment of epilepsy, anxiety, and infantile convulsions (Azmi et al., 2011; Bum et al., 2004; Majeed et al., 2021; Tripathi et al., 2022). Previous studies indicated that the decoction prepared from the leaves of *Mimosa pudica* administered orally to mice strongly protected against pentylenetetrazol- and strychnine-induced generalised clonic–tonic convulsions. It also had a potent anticonvulsant property against the turning behaviour and *exitus* induced by *N*-methyl-D-aspartate. Based on its traditional uses and previous findings, it should be interesting to investigate whether the *Mimosa pudica* aqueous extract could protect animals against epileptogenesis of epilepsy and, eventually, temporal lobe epilepsy (Bum et al., 2004). In addition, previous phytochemical studies showed that the *Mimosa pudica* aqueous extract contains some bioactive components such as terpenoids, flavonoids (quercetin-7-rhamnoside, luteolin 3, acacetin-7-rutinoside, and quercetin-3-glucoside-7-rhamnoside), glycosides, alkaloids, quinines, phenols, tannins, flavonoids, and saponins. Other studies demonstrated the presence of mimosine, crocetin dimethyl ester, fatty acids, and green oil (Joseph et al., 2013; Kiruba et al., 2011). The *Mimosa pudica* extract exerts anti-inflammatory, antinociceptive, and analgesic activities in rodents (Joseph et al., 2013; Khalid et al., 2011). Other studies have established antihyperglycemic, antivenomous, immunomodulatory, antihepatotoxic, diuretic, and antimalarial activities (Azmi et al., 2011; Baghel et al., 2013; Ganguly et al., 2007). However, no scientific evidence has been reported about the antiepileptogenic and anticonvulsant properties of *Mimosa pudica* extracts in the mouse model of temporal lobe epilepsy. Therefore, we hypothesised that the *Mimosa pudica* aqueous extract could antagonise *status epilepticus*, epileptogenesis, and epileptic seizures in this pharmacoresistant model. The overall objective of this research was to evaluate the anticonvulsant and antiepileptogenic properties of an aqueous extract of *Mimosa pudica* in the pilocarpine–picrotoxin model of temporal lobe epilepsy and investigate the effects of the aqueous extract of *Mimosa pudica* leaves and stems on some parameters of *status epilepticus* and epileptic convulsions, oxidative and nitrosative stresses, GABAergic and cholinergic transmissions, and the expression of BDNF.

## 2 Materials and methods

### 2.1 Plant material and preparation of the *Mimosa pudica* aqueous extract

The leaves and stems of *Mimosa pudica* used in our study were harvested in Buea (July 2021), Fako division (the Southwest Region of Cameroon; harvesting coordinates 4°15′06″N and 9°29′03″E). The field studies did not involve protected species. The plant sample was authenticated by the National Herbarium of Yaoundé (Cameroon), where a voucher was deposited (sample number 54102/HNC).

The aqueous extract of *Mimosa pudica* leaves and stems was prepared by using a method similar to that of Cameroonian traditional healers. The leaves and stems of *Mimosa pudica* are the preferred part of the plant used in Cameroonian traditional medicine for treating epilepsy and infantile convulsions. According to traditional healers, the leaves and stems are usually harvested, sun dried, and pulverized to obtain powder. Approximately 100 g of the powdered material is macerated in 500 mL of water and boiled. The decoction obtained is administered orally to epileptic patients at the dose range of 40–160 mg/kg during or before the occurrence of epileptic seizures. Therefore, in our experiments, the leaves and stems of *Mimosa pudica* were cut into pieces and allowed to dry at room temperature (25°C). The dried leaves and stems were then reduced to fine particles. The powder (500 g) was boiled in 5,000 mL of distilled water for 20 min. After it cooled, the concoction was filtered with Whatman No. 1 filter paper. The collected filtrate (aqueous extract) was dried using a rotary evaporator. The aqueous extract of *Mimosa pudica* leaves and stems was prepared using distilled water at a concentration of 16 mg/mL and considered an initial concentration. The extract was prepared daily, 45 min to 1 h before its oral administration to mice using a non-flexible gavage needle with round end, and fixed at the extremity of a 1-mL syringe. The aqueous extract of *Mimosa pudica* leaves and stems was given 1 h before each pharmacological testing at a volume of 10 mL/kg, and the concentrations 2, 4, 8, and 16 mg/mL were used, respectively.

### 2.2 High-performance liquid chromatography analysis of the aqueous extract of *Mimosa pudica*

An amount of 10 mg of the sample (*Mimosa pudica* aqueous extract) was dissolved in 10 mL methanol. The solution was filtered through a membrane filter prior to high-performance liquid chromatography analysis. The sample was screened by means of an HPLC system (AKTA™ Purifier, Amersham Biosciences). The aqueous extract of *Mimosa pudica* was analysed by HPLC using a Vydac C18 column (4.6 × 250 mm, 5 μm particle size), and for elution of the compounds, a gradient of two solvents denoted A and B was employed. The mobile phases were 90% acetonitrile mixed with water (called A) and 0.1% trifluoroacetic acid mixed with water (called B). The flow rate used for this experiment was set up at 1.0 mL/min and used a volume of 10 µL as the injection volume. The retention time and UV spectrum of major peaks were analysed and compared with standard compounds. The eluant was monitored at 215 nm. Finally, the fractions eluted from the HPLC system were then combined in tubes and then lyophilized to afford the products as powders.

### 2.3 Chemicals and reagents

Acetylthiocholine iodide, Ellman’s reagent, pilocarpine hydrochloride, picrotoxin, sodium valproate, vitamin C, NaCl, Tris-HCl, trichloroacetic acid, thiobarbituric acid and all the other reagents used for biochemical determination were purchased from Sigma-Aldrich, St Louis, United States.

### 2.4 Experimental animals and ethical consideration

Adult male mice, *Mus musculus* Swiss, weighing 25 ± 2 g, 2–3 months old, were used in this experiment. They were obtained from the National Veterinary Laboratory, Garoua, Cameroon, and acclimatized for 3 days (72 h) in the Life Science Laboratory of the University of Buea, Cameroon. The animals were housed in conventional cages (45 cm long, 45 cm wide, 25 cm high) at 25°C, on a 12/12-h light–dark cycle, with lights on at 06:00 h and off at 18:00 h. They were supplied with food and water *ad libitum*. All experiments were performed according to the Guide for the Care and Use of Laboratory Animal published by the United States National Institutes of Health (NIH publication No. 85-23, revised 1996). In addition, all the experiments were approved by the University of Buea–Institutional Animal Care and Use Committee (UB-IACUC) with the following permit number: UB-IACUC N 07/2022. All efforts were made to minimize animal suffering and reduce the number of animals used.

### 2.5 Pharmacological testing

#### 2.5.1 Acute pilocarpine-induced *status epilepticus* test

**FIGURE 1:**
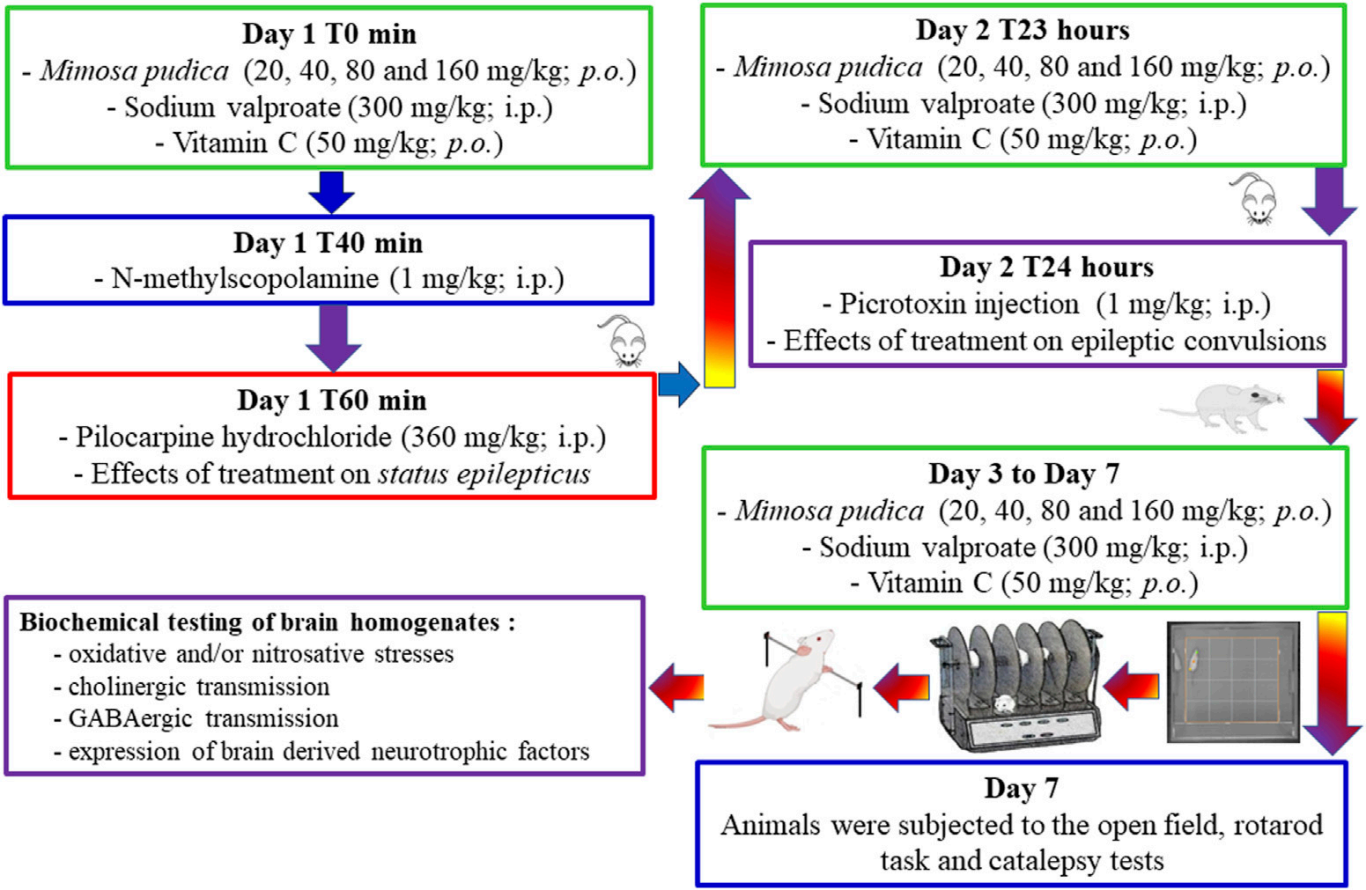
Schematic representation of the experimental design.

Mice were randomly grouped in different lots of six mice each and administered the following treatment: groups 1 (normal control group) and 2 (negative control group) received orally 10 mL/kg of distilled water; groups 3–6 received orally different doses of the *Mimosa pudica* aqueous extract (test groups; 20, 40, 80, and 160 mg/kg); and group 7 received sodium valproate (positive control group 1; 300 mg/kg). Forty minutes after the first administration of the different treatments to mice, they were injected intraperitoneally with N-methylscopolamine at a dose of 1 mg/kg to lower the peripheral effects of pilocarpine. Twenty minutes after the injection of N-methyl-scopolamine, *status epilepticus* was induced in each mouse by an acute intraperitoneal injection of 360 mg/kg pilocarpine (Moto et al., 2018; Nguezeye et al., 2023). Mice from the normal control group named group 1 did not receive N-methylscopolamine and pilocarpine; however they received saline. Immediately after the intraperitoneal injection of pilocarpine, each animal was returned to its cage, and their behaviour was observed for a 6-h duration. Severity and duration of acute epileptic seizures were categorised based on the Racine scale (Phelan et al., 2015). Hypoactivity, followed by behavioural changes (orofacial modifications, twitching of vibrissae, yawning, salivation, eye blinking, and tonic–clonic seizures), was observed in the studied animals. During the experiment, animals were video-monitored for the appearance of *status epilepticus*. The severity of seizures was assessed using the Racine scale: stage 0: no response; stage 1: hyperactivity and clonus of vibrissae; stage 2: shaking of the head and myoclonic jerks; stage 3: unilateral clonus of the forelimbs; stage 4: rearing and bilateral clonus of the forelimbs; stage 5: tonic–clonic seizures with the loss of the righting reflex. The animals were selected for further studies (day-second) based on the development of stage 5 attack of the Racine scale for two consecutive periods (Figure 1).

#### 2.5.2 Anticonvulsant properties of *Mimosa pudica* aqueous extracts during the acute phase of temporal lobe epilepsy induced by picrotoxin in pilocarpine-treated mice

*Excitus* and convulsions were induced by picrotoxin in mice 24 h after the acute pilocarpine-induced *status epilepticus* test. This spontaneous neuropathology was facilitated by intraperitoneal injection of a sub-convulsive dose of picrotoxin (1 mg/kg) to mice. In brief, animals were treated for the second day, or 23 hours after the injection of pilocarpine, with distilled water for groups 1 and 2, the respective doses of the extracts for groups 3–6, and sodium valproate for group 7, respectively. One hour later, a sub-convulsive dose of picrotoxin (1 mg/kg) was injected intraperitoneally to mice (groups 2–7), except group 1 that was injected intraperitoneally with saline. Each animal was observed immediately for a period of 30 min, and the incidence of seizures (the latency time to first clonic seizure, latency time to first tonic seizure, the number of clonic seizures, duration of clonic seizures, the number of tonic seizures, and the duration of tonic seizures) was noted (Hamani and Mello, 1997; Nguezeye et al., 2023). Tonic–clonic seizures involve both tonic (a sudden stiffness or tension in the muscles of the arms, legs, or trunk) and clonic (twitching or hock-like jerks of a muscle or a group of muscles) phases of muscle activity. The latency of tonic–clonic seizures was used to determine the seizure score. This score was calculated according to the following formula: Score = 1- negative control group latency/test group or positive control group latency (Taiwe et al., 2016b).

#### 2.5.3 Behavioural assessment

On day 7, mice were grouped into different lots of six mice each and administered the following treatment: groups 1 (normal control group) and 2 (negative control group) received 10 mL/kg of distilled water orally; groups 3–6 received different doses of the *Mimosa pudica* aqueous extract orally (test groups; 20, 40, 80, and 160 mg/kg); and group 7 received sodium valproate (positive control group 1; 300 mg/kg). One group of naïve mice (not pilocarpine-challenged mice) was added and treated orally with 50 mg/kg vitamin C, which served as a reference drug (positive control group) for the behavioural tests (exploration, locomotion, and motor coordination) (Martinc et al., 2014). One hour after the last daily administration of the different doses of the plant extracts or the standard drugs, mice were observed for 5 min in the open-field paradigm (day 7), 1 min on the rotating rod (day 7), and 5 min on a horizontal bar (day 7) (Figure 1).

##### 2.5.3.1 Open-field test

Locomotor activity, exploration, and ambulatory behaviour were quantified by using the open-field paradigm (a wooden square box: 40 cm × 40 cm × 45 cm; with 16 smaller squares: 10 cm × 10 cm). Each animal was placed individually in the centre of the arena and allowed to explore the open arena freely (Taïwe et al., 2012). Each animal was observed in the open field to score crossing (the number of square floor units entered), rearing (the number of times the animal stood on its hind legs), grooming, defecation, and centre time, 1 h after administration of *Mimosa pudica* aqueous extracts (20, 40, 80, and 160 mg/kg), sodium valproate (300 mg/kg, i.p.), vitamin C (50 mg/kg, orally), or vehicle (10 mL/kg, p.o.). Mice were video-tracked and recorded using ANY-maze version 6.03 (Stoelting Company, Wood Dale, IL, United States). At the end of each experiment, faecal boli defecated was collected, and the entire apparatus was wiped with 95% ethanol prior to use and before subsequent tests to remove any scent clues left by the previous subject mouse.

##### 2.5.3.2 Rotarod test

On day 7, 15 min after the open-field paradigm, the motor coordination test was used to measure any sign of neurotoxicity and locomotion of treated mice using the rotating rod method. An initial selection of animals was performed on the previous day of experiment excluding those that did not remain on the rotarod bar during a 1-min session each. The bar with a diameter of 2.50 cm was rotated at a constant speed of 12 revolutions per minute (Figure 1). The integrity of motor coordination was measured on the basis of the number of falls from the revolving bar in 1 min. During the test session itself, that is, after the oral administration of the different treatments, both the latency to fall from the rotating rod and the number of falls were determined (Taiwe et al., 2016a).

##### 2.5.3.3 Catalepsy test

On day 7, 15 min after the rotarod test, catalepsy was evaluated according to the standard bar hanging procedure by placing each animal with both forelegs over a horizontal bar, elevated 4.5 cm from the floor (Waku et al., 2021). Catalepsy was considered finished when the forepaw touched the floor or when the animal climbed the bar. Measurement was done for a duration of 30 and 60 min 15 min after the rotarod test. The time during which the animal maintained the cataleptic position was determined for up to 5 min, with three attempts allowed to replace the mouse over the bar.

#### 2.5.4 Biochemical analysis

Immediately after the last behavioural test, each animal was sacrificed; the brain was dissected and cleaned using the ice-cold saline solution (0.9%, w/v). The hippocampus was removed and weighed. Homogenates (10%, w/v) were prepared with ice-cold 0.1 M phosphate buffer (pH 7.4) and centrifuged at 10,000 rpm for 15 min duration. The aliquots of the obtained supernatants were then collected and used to determine, according to different protocols, the level of each studied biochemical parameters.

##### 2.5.4.1 Evaluation of the brain GABA concentration and determination of GABA-transaminase activity

Concentration of GABA in the brain homogenate was quantified as described previously (Sandoval-Salazar et al., 2016; Vega Rasgado et al., 2018), and the final GABA level in each homogenate was expressed in µg/g of wet tissue (Taïwe et al., 2010). The GABA-transaminase (GABA-T) activity was measured in the homogenates using the spectrophotometric method (Szyndler et al., 2006; Vega Rasgado et al., 2018).

##### 2.5.4.2 Quantification of nitrosative and oxidant stress markers

Malondialdehyde (MDA), an indicator of lipid peroxidation, was determined with a spectrophotometer using thiobarbituric acid assay (Liu et al., 2012). Reduced glutathione (GSH, endogenous antioxidant) was quantified by its reaction with 5, 5′-dithiobis (2- nitrobenzoic acid) (Ellman’s reagent) to yield a yellow chromophore (Rahman et al., 2006). The activity of superoxide dismutase (SOD) was assayed according to the method described by Sun et al. (Mesa-Herrera et al., 2019), while the nitrite level (NO) (indicator of nitric oxide production) was estimated using the method of Cortas and Wakid (Filgueiras et al., 2021).

##### 2.5.4.3 Determination of the brain cholinergic status

Acetylcholinesterase (AChE) and butyrylcholinesterase (BChE) were quantified in the hippocampal homogenates, according to the method of Ellman (Ni et al., 2016). The kinetic profile of enzyme cholinesterase activities was determined using a spectrophotometer at 412 nm for 3 min at 30-s intervals. One unit of acetylcholinesterase or butyrylcholinesterase activity was defined as the number of micromoles (μmol) of acetylthiocholine iodide or butyrylthiocholine iodide hydrolysed per min per mg of protein.

##### 2.5.4.4 Measurement of BDNF

The concentration of BDNF was determined using the Promega BDNF Emax^®^ ImmunoAssay System kit (Madison, United States), as indicated by the manufacturer’s protocol. In brief, the hippocampus was weighed, homogenized in lysis buffer, and centrifuged (12,000 × g, 4°C) for 5 min; then, the supernatants were collected. The relative concentration of BDNF was expressed as per milligram total protein in the tissue. Protein was measured as described previously (Augustyniak et al., 2015), using bovine serum albumin as the standard and measured in the range of 0.01–0.10 mg/mL.

### 2.6 Acute toxicity studies of the *Mimosa pudica* aqueous extract in naïve mice

The acute toxicity of the *Mimosa pudica* aqueous extract was determined using an established guideline (Abubakar et al., 2022; Erhirhie et al., 2018). The extract was given orally, at a dose of 5,000 mg/kg, to one female mouse under fasting for 8 h. Thereafter, the same dose of extract was administered to four female mice, giving a total of five animals at intervals of 48 h. One normal group of animals, which constituted of five female mice, received distilled water (10 mL/kg). Each animal was observed for 24 h immediately after the administration of the treatment, and then, they were further observed for up to 14 days for any signs of toxicity and deaths, as well as for the latency of death. Finally, the global harmonised system was used for the estimation of the lethal dose 50 (DL_50_) of the *Mimosa pudica* aqueous extract.

### 2.7 Statistical analysis

Eight groups of animals were used in this study (seven groups for the anticonvulsant test and one group of animals were added during behavioural testing). Results were expressed as means ± standard error of mean (SEM), for six animals per group; statistical differences between controls and treated groups were tested by a one-way analysis of variance (ANOVA), followed by Tukey’s multiple comparison test. The differences were considered significant at P < 0.05. Statistical analyses were performed using GraphPad prism 9.0.0 (GraphPad Software, San Diego, CA, United States).

## 3 Results

### 3.1 Yield of extraction of the *Mimosa pudica* aqueous extract

After it cooled, the decoction prepared from *Mimosa pudica* leaves and stems was filtered with Whatman No. 1 filter paper. The collected filtrate (aqueous extract) was dried using a rotary evaporator, and a dry extract was obtained [yield of the extraction was 10.20% (w/w)].

### 3.2 High-performance liquid chromatography profile of the *Mimosa pudica* aqueous extract

**FIGURE 2:**
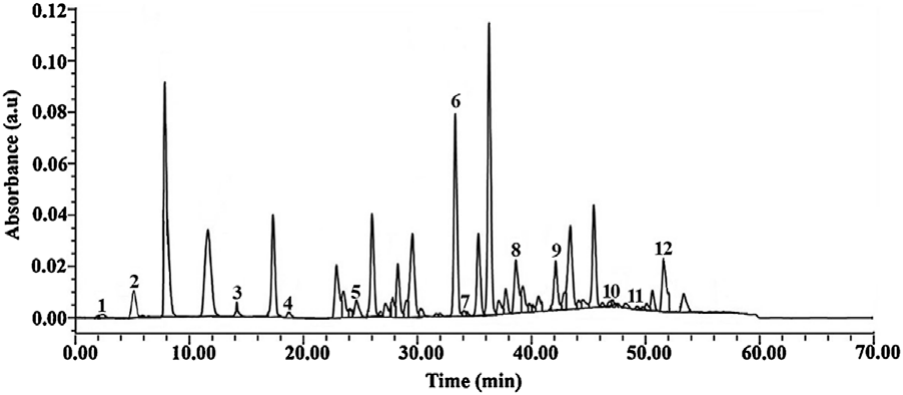
HPLC chromatogram of the *Mimosa pudica* aqueous extract showing peaks with similar retention time to the eluted compounds. 1: Gallic acid (2.15 min), 2: chlorogenic acid (5.09 min), 3: ferulic acid (14. 05 min), 4: hyperoside (18.34 min), 5: luteolin (26.89 min), 6: fisetin (33.47 min) 7: apigenin7-glucoside (34.01 min), 8: naringenin (38.22 min), 9: benzene-triol (42.15 min), 10: apigenin (47.09 min), 11: chrysin (49.36 min), and 12: mimosine (51.83 min).

The HPLC chromatograms of the *Mimosa pudica* aqueous extract showed the presence of some active compounds, and the majority of the components were eluted between 2.15 and 51.83 min, corresponding to 0.01–0.12 AU (Figure 2). The number of HPLC peaks (fractions) collected from the aqueous extract of *Mimosa pudica* was 12, corresponding respectively to 12 chemical compounds. When compared with standard compounds, some of them were elucidated: gallic acid (1), chlorogenic acid (2), ferulic acid (3), hyperoside (4), luteolin (5), fisetin (6) apigenin7-glucoside (7), naringenin (8), benzene-triol (9), apigenin (10) chrysin (11), and mimosine (12) (Figure 2).

### 3.3 Effects of the *Mimosa pudica* aqueous extract on *status epilepticus* and epileptic convulsions induced by pilocarpine and picrotoxin

**FIGURE 3:**
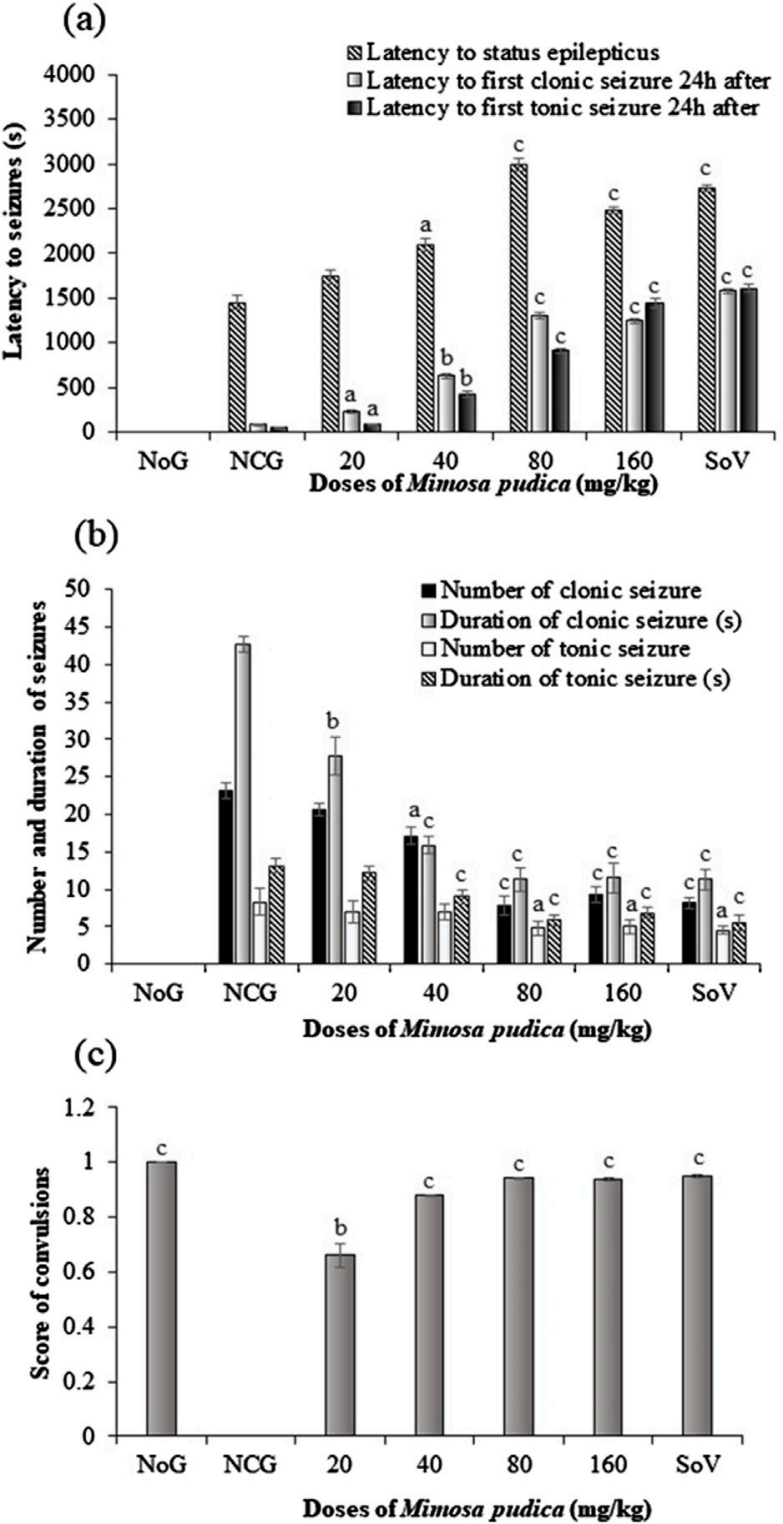
Effects of the *Mimosa pudica* aqueous extract on *status epilepticus* and convulsions induced by pilocarpine and picrotoxin (latency to *status epilepticus*, first clonic and tonic–clonic seizures 24 h after *status epilepticus* **(A)**, number and duration of clonic, tonic–clonic seizures **(B)**, and the score of generalized tonic–clonic seizures **(C)**. Results are expressed as mean ± S.E.M. n = 6 animals. Statistical differences were tested using one-way ANOVA, followed by Tukey’s test (HSD) multiple comparison test. Significant difference set at ^a^P < 0.05, ^b^P < 0.01, and ^c^P < 0.001, versus the negative control group. NoG, normal group treated with distilled water; NCG, negative control group treated with distilled water; SoV, sodium valproate (300 mg/kg); s, second. All mice were subjected to epileptogenesis induced by pilocarpine (360 mg/kg) except the normal group.

Oral administration of the *Mimosa pudica* aqueous extract produced a significant difference [F (6, 35) = 14.01, p < 0.001] in the latency time to *status epilepticus* in mice (Figure 3A). The extract significantly increased the latency to *status epilepticus* from 1,451.00 ± 83.61 s in the distilled water-treated pilocarpine–picrotoxin group to 2,997.40 ± 72.45 s (P < 0.001) and 2,479.95 ± 34.83 (P < 0.001) s, in the tests administered 80 and 160 mg/kg, respectively. Similarly, 300 mg/kg sodium valproate significantly (P < 0.001) increased the latency to *status epilepticus* from 1,451.00 ± 83.61 s to 2,732.00 ± 35.81 s (P < 0.001) in the positive control group of mice.

One-way ANOVA revealed a significant difference in the effect of *Mimosa pudica* and pilocarpine–picrotoxin treatments [F (6, 35) = 52.59 p < 0.001] in the latency to first clonic seizure 24 h after *status epilepticus*. *Mimosa pudica* administration significantly (P < 0.001) delayed the latency to first clonic seizures to 1,301.54 ± 29.42 s (P < 0.001) and 1,243.04 ± 23.31 s for the groups of animals treated with the doses of 80 and 160 mg/kg, respectively, when compared with the negative control group (83.33 ± 7.45 s) (Figure 3A).

The standard antiepileptic drug like the *Mimosa pudica* extract significantly (p < 0.001) increased this latency time, as compared to the vehicle-treated pilocarpine–picrotoxin mice (Figure 3A). As shown in Figure 3A, one-way ANOVA revealed a significant effect of treatments [F (6, 35) = 47.91, P < 0.01] in the latency to the first clonic seizure 24 h after *status epilepticus*. Moreover, pilocarpine–picrotoxin injection significantly reduced this latency time to 50 ± 3.29 s in comparison with the normal control group of mice. However, pre-treatment of mice with the 40–160 mg/kg *Mimosa pudica* aqueous extract significantly increased the latency to first clonic seizure to 906.50 ± 31.78 s and 1,443.67 ± 50.87 s, respectively when compared with vehicle-treated pilocarpine–picrotoxin (Figure 3A).

One-way ANOVA revealed a significant effect of treatment in the number of clonic seizures [F (6, 35) = 477 p < 0.001] and duration [F (6, 35) = 466 p < 0.001] (Figure 3B). The number of clonic seizures decreased from 23.16 ± 0.98 in the negative control group to 7.83 ± 1.16 (P < 0.001) and 9.33 ± 1.03 (P < 0.001) in the groups of mice administered 80 and 160 mg/kg extract, respectively (Figure 3B). The duration of clonic seizure also decreased from 42.66 ± 1.03 s in the negative control group to 11.33 ± 1.63 s (P < 0.001) and to 11.55 ± 2.07 s (P < 0.001) in the group of mice administered with the doses of 80 and 160 mg/kg aqueous extract, respectively. Likewise, sodium valproate (300 mg/kg) decreased the number of clonic seizure and duration to 8.16 ± 0.75 (P < 0.001) s and 11.33 ± 1.36 s (P < 0.001), respectively, when compared to the vehicle-treated pilocarpine–picrotoxin mice (Figure 3B).

As shown in Figure 3B, one-way ANOVA revealed a significant effect of treatments for the number [F (6, 35) = 36.95 p < 0.001] and duration [F (6, 35) = 168.6 p < 0.001] of clonic seizures. The *Mimosa pudica* aqueous extract significantly reduced the number of clonic seizures form 8.33 ± 1.75 in the pilocarpine–picrotoxin model to 4.83 ± 0.98 (P < 0.001) and 5.00 ± 0.89 in the groups of mice administered 80 and 160 mg/kg aqueous extract, respectively. Interestingly, the extract also reduced the duration of clonic seizures from 13.16 ± 1.98 s in the pilocarpine–picrotoxin model to 5.83 ± 0.75 s (P < 0.001) and 6.83 ± 0.75 s (P < 0.001) at the dose 80 and 160 mg/kg *Mimosa pudica* extract, respectively. Obviously, the standard antiepileptic drug, sodium valproate (300 mg/kg), reduced the number of clonic seizure from 8.33 ± 1.75 s in the negative control group to 4.50 ± 0.54 s (P < 0.001) and its corresponding duration from 13.16 ± 0.98 s in the pilocarpine model to 5.50 ± 1.04 s (P < 0.001) (Figure 3B).

One-way ANOVA indicated a significant effect of treatments [F (6, 35) = 2,840 p < 0.001] in the score of clonic seizure. The score of clonic seizure increased from 0 in the distilled water-treated pilocarpine–picrotoxin mice to 0.92 ± 0.01 (P < 0.001) and 0.94 ± 0.01, in the groups administered 80 and 160 mg/kg *Mimosa pudica* respectively. Similarly, the antiepileptic drug sodium valproate (300 mg/kg) significantly increased (P < 0.001) the score of clonic seizures when compared to the negative control group (Figure 3C).

### 3.4 Effects of the *Mimosa pudica* aqueous extract on locomotion and exploratory behaviour

#### 3.4.1 Effects of the *Mimosa pudica* aqueous extract on exploratory behaviour in the open-field test

As reported below (Table 1), administration of pilocarpine–picrotoxin in the negative control group of mice caused a reduction in the number of crossing, grooming, and time spent at the centre of the open field in comparison with those of the normal group. The *Mimosa pudica* aqueous extract increased the crossing (P < 0.001), grooming (P < 0.01), and time spent at the centre of the open field (P < 0.001) in the test groups treated with the plant extract. Sodium valproate (300 mg/kg, i.p.) and vitamin C (50 mg/kg, p.o.) also induced an increase in the crossing, grooming, and time spent in the centre. The number of rearing (P < 0.001) and faecal boli (P < 0.001) decreased when compared with the negative control groups, respectively. Likewise, mice treated with sodium valproate (300 mg/kg, i.p.) and vitamin C (50 mg/kg, p.o.) also expressed significant performance comparable to that of *Mimosa pudica*-treated pilocarpine–picrotoxin mice (Table 1).

**TABLE 1:** Effects of the *Mimosa pudica* aqueous extract on rearing, crossing, grooming, centre time, and quantity of faecal boli in the mouse activity of pilocarpine–picrotoxin-treated animals subjected to the open-field test.

Parameter	NoG	NCG	*Mimosa pudica* (mg/kg)				SoV (mg/kg)	VitC (mg/kg)
			20	40	80	160	300	50
Rearing	6.33 ± 1.00^a^	10.83 ± 0.88	4.16 ± 0.83^b^	3.66 ± 0.77^b^	2.33 ± 0.66^c^	1.83 ± 0.55^c^	1.33 ± 0.44^c^	1.50 ± 0.66^c^
Crossing	9.16 ± 0.88^b^	4.16 ± 0.83	14.16 ± 1.88^c^	19.83 ± 1.16^c^	26.83 ± 1.88^c^	32.33 ± 1.33^c^	34.16 ± 1.55^c^	32.16 ± 5.83^c^
Grooming	2.66 ± 0.44^a^	1.16 ± 0.27	2.33 ± 0.44^a^	3.16 ± 0.55^b^	3.83 ± 0.88^b^	4.16 ± 0.88^b^	4.16 ± 0.83^b^	4.33 ± 0.66^b^
Faecal boli (g)	0.21 ± 0.06^b^	0.64 ± 0.13	0.19 ± 0.02^b^	0.06 ± 0.06^c^	0.04 ± 0.05^c^	0.03 ± 0.05^c^	0.03 ± 0.04^c^	0.03 ± 0.04^c^
Centre time (s)	8.33 ± 1.11^a^	3.83 ± 0.27	6.33 ± 1.55^a^	21.16 ± 5.55^c^	23.66 ± 1.55^c^	27.66 ± 1.22^c^	30.33 ± 1.33^c^	35.66 ± 5.22^c^

Results are expressed as mean ± S.E.M. n = 6 animals. Statistical differences were tested using one-way ANOVA, followed by Tukey’s test (HSD). Significant difference set at ^a^P < 0.05, ^b^P < 0.01, and ^c^P < 0.001, versus the negative control group. NoG, normal group; NCG, negative control group treated with distilled water (10 mL/kg) and pilocarpine (360 mg/kg); SoV, sodium valproate (300 mg/kg); VitC, vitamin C (50 mg/kg).

#### 3.4.2 Effects of the *Mimosa pudica* aqueous extract on locomotion in the rotarod test

A significant difference in the motor coordination of different groups of treated mice is shown in Table 2 with respect to time, that is, at 0 min [F (7, 40) = 19.88; P < 0.05], 30 min [F (7, 40) = 62.14; P < 0.01], and 60 min [F (7, 40) = 142.1; P < 0.001]. The time spent on the rotating bar in the normal group of mice was significantly higher than that seen in the negative control group at 0 min, 30 min, and 60 min. Similarly, the aqueous extracts of *Mimosa pudica* administered at the doses of 80 and 160 mg/kg significantly prolonged the time spent on the rotating bar when compared to the time registered in the negative control group (Table 2). In addition, the plant extract significantly reduced the number of falls at 0 min (P < 0.001), 30 min (P < 0.001), and 60 min (P < 0.001), respectively, when compared with the negative control. The level of the number of falls in the distilled water-treated pilocarpine–picrotoxin mice was significantly higher than that observed in the distilled water-treated mice. The doses of 80 and 160 mg/kg reduced the fall at 30 and 60 min when compared to the negative control group. Likewise, mice treated with sodium valproate (300 mg/kg, i.p.) and vitamin C (50 mg/kg, *p.o.*) also expressed remarkable performance comparable to that of *Mimosa pudica*-treated pilocarpine–picrotoxin mice (Table 2).

**TABLE 2:** Effects of the *Mimosa pudica* aqueous extract on the locomotor activity of pilocarpine–picrotoxin-treated animals subjected to the rotarod test.

Treatment (mg/kg)	NoG	NCG	*Mimosa pudica* (mg/kg)				SoV (mg/kg)	VitC (mg/kg)
	**--**		20	40	80	160	300	50
Time on the bar								
0 min	52.55 ± 1.88^b^	22.16 ± 4.83	35.66 ± 5.22	39.16 ± 3.16	46.33 ± 4.66^a^	48.66 ± 2.66^a^	49.33 ± 7.44^a^	52.83 ± 2.88^b^
30 min	55.33 ± 2.33^b^	20.16 ± 2.83	40.16 ± 2.16^a^	46.16 ± 3.22^a^	48.83 ± 3.50^a^	52.83 ± 3.16^b^	54.16 ± 2.22^b^	55.66 ± 2.33^b^
60 min	60.00 ± 0.00^c^	15.16 ± 0.83	49.16 ± 6.16^c^	56.33 ± 4.22^c^	59.83 ± 0.27^c^	60.00 ± 0.00^c^	60.00 ± 0.00^c^	60.00 ± 0.00^c^
Number of falls								
0 min	0.33 ± 0.44^c^	3.83 ± 0.55	1.66 ± 0.44^b^	1.83 ± 0.55^b^	1.33 ± 0.44^b^	1.16 ± 0.27^c^	1.16 ± 0.27^c^	1.16 ± 0.27^c^
30 min	1.16 ± 0.27^c^	3.66 ± 0.77	1.61 ± 0.66^b^	1.33 ± 0.55b	1.16 ± 0.27^c^	1.16 ± 0.61^c^	0.83 ± 0.27^c^	0.83 ± 0.27^c^
60 min	0.00 ± 0.00^c^	2.50 ± 0.50	1.33 ± 0.44^b^	0.50 ± 0.50^c^	0.16 ± 0.27^c^	0.00 ± 0.00^c^	0.00 ± 0.00^c^	0.00 ± 0.00^c^

Results are expressed as mean ± S.E.M. n = 6 animals. Statistical differences were tested by a one-way ANOVA, followed by Tukey’s (HSD) multiple comparison test; the differences were considered significant at ^a^P < 0.05, ^b^P < 0.01, and ^c^P < 0.001, versus the negative control group. NoG, normal group; NCG, negative control group treated with distilled water (10 mL/kg) and pilocarpine (360 mg/kg); SoV, sodium valproate (300 mg/kg); VitC, vitamin C (50 mg/kg).

#### 3.4.3 Effects of the *Mimosa pudica* aqueous extract on motor coordination in the catalepsy test

**FIGURE 4:**
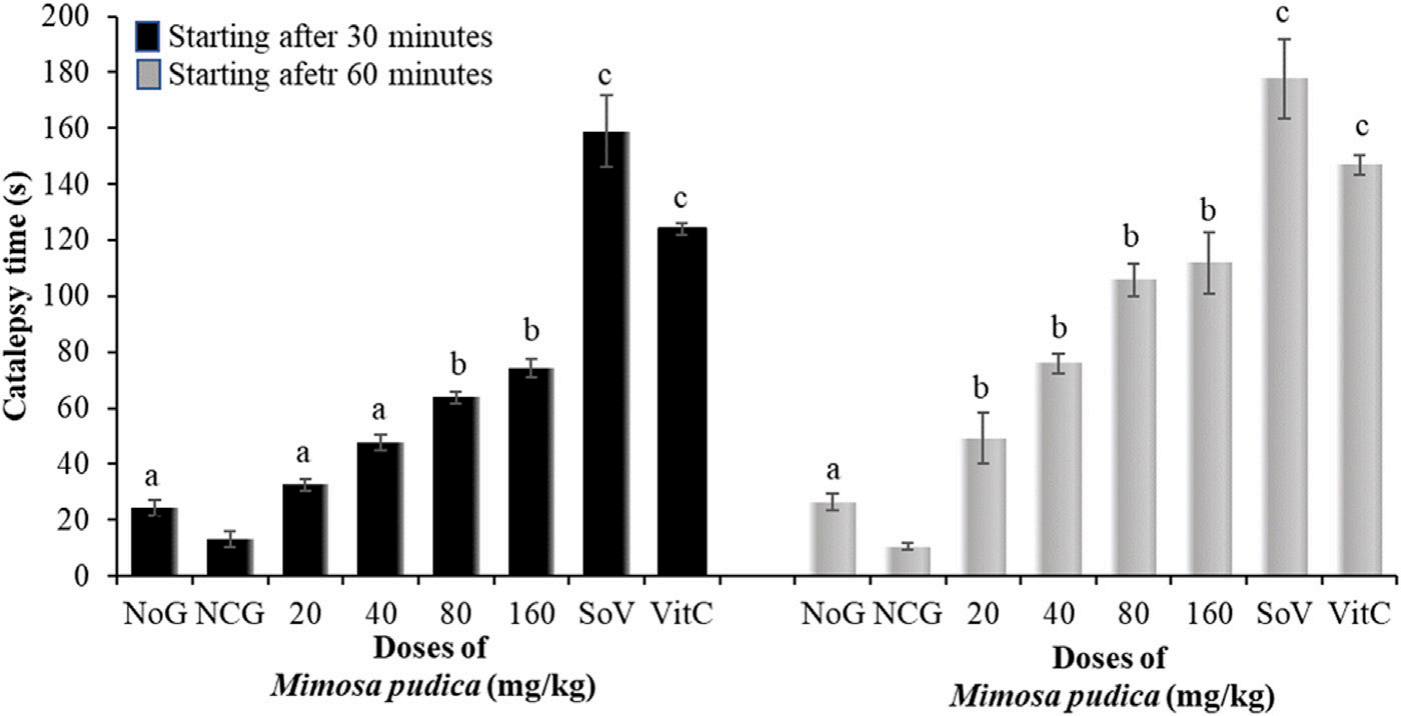
Effects of the *Mimosa pudica* aqueous extract on the motor coordination of pilocarpine–picrotoxin-treated mice subjected to the catalepsy test. Results are expressed as mean ± S.E.M. n = 6 animals. Statistical differences were tested by a one-way ANOVA, followed by Tukey’s (HSD) multiple comparison test; the differences were considered significant at ^a^P < 0.05, ^b^P < 0.01, and ^c^P < 0.001, versus the negative control group. NoG, normal group treated with distilled water; NCG, negative control group treated with distilled water; SoV, sodium valproate (300 mg/kg); VitC, vitamin C (50 mg/kg); s, second. All mice were subjected to epileptogenesis induced by pilocarpine (360 mg/kg) except the normal group.

Analysis of variance depicted a significant effect of oral administration of *Mimosa pudica* in pilocarpine-treated mice subjected to the catalepsy test at 30 min [F (7, 56) = 239.9; P < 0.001] and 60 min [F (7, 56) = 350.7; P < 0.001]. There was a significant reduction of catalepsy between the normal groups and the negative control group. *Mimosa pudica*, when administered at the respective doses 80 and 160 mg/kg, triggered catalepsy at 30 min (63.75 ± 2.25 and 74.25 ± 3.31 s) and 60 min (105.74 ± 6.00 and 111.87 ± 10.81 s) in animals (Figure 4). The effects of *Mimosa pudica* were similar to those of 300 mg/kg sodium valproate (158.75 ± 12.81 and 177.62 ± 14.37 s, respectively, at 30 and 60 min) and vitamin C (50 mg/kg, given orally; 124.12 ± 2.12 and 146.87 ± 3.62 s, respectively, at 30 and 60 min) (Figure 4).

### 3.5 Effects of the *Mimosa pudica* aqueous extract on biochemical parameters

#### 3.5.1 Effects of the *Mimosa pudica* aqueous extract on the level of GABA and the activities of GABA-transaminase, acetylcholinesterase, and butyrylcholinesterase

A significant difference was observed in the levels of GABA in the hippocampus of all groups of mice [F (7, 40) = 273.3; P < 0.01] (Table 3). GABA levels were significantly lower in the negative control group than those observed in the normal group. The plant extract significantly increased this concentration by 40.15% and 47.23% at the doses of 80 and 160 mg/kg, respectively. Obviously, sodium valproate and vitamin C also significantly increased the level of GABA by 46.74% and 47.62% in the hippocampus, at the doses of 300 and 50 mg/kg, respectively.

**TABLE 3:** Effects of the *Mimosa pudica* aqueous extract on GABAergic and cholinergic neurotransmission in the hippocampus of pilocarpine–picrotoxin-treated mice.

Treatment (mg/kg)	NoG	NCG	*Mimosa pudica* (mg/kg)				SoV (mg/kg)	VitC (mg/kg)
	**--**		20	40	80	160	300	50
GABA (μg/g de tissue)	394.67 ± 1.33^b^	265.67 ± 6.67	273.17 ± 4.17	304.83 ± 4.11^a^	372.33 ± 10.67^b^	391.17 ± 4.83^b^	389.83 ± 5.56^b^	392.17 ± 5.06^b^
GABA-T (pg/min/mg de tissue)	42.33 ± 0.44^b^	105.83 ± 5.17	79.67 ± 4.89^a^	68.83 ± 7.17^a^	54.83 ± 3.5^b^	42.33 ± 3.89^b^	40.17 ± 1.83^b^	45.17 ± 1.89^b^
AchE (µmol/min/mg de tissue)	12.51 ± 1.08^a^	16.61 ± 0.68	15.42 ± 0.58	13.37 ± 0.93^a^	12.33 ± 1.03^a^	12.37 ± 0.93^a^	12.87 ± 0.88^a^	12.05 ± 0.80^a^
BchE (µmol/min/mg of tissue)	11.87 ± 0.87^a^	17.54 ± 0.75	15.87 ± 0.91	13.89 ± 1.01^a^	11.638 ± 0.63^a^	11.93 ± 0.78^a^	11.92 ± 1.03^a^	11.01 ± 0.53^a^

Results are expressed as mean ± S.E.M. n = 6 animals. Statistical differences were tested using one-way ANOVA, followed by Tukey’s test (HSD). Significant difference set at ^a^P < 0.05, ^b^P<0.01, and ^c^P < 0.001, versus the negative control group. NoG, normal group; NCG, negative control group treated with distilled water (10 mL/kg) and pilocarpine (360 mg/kg); SoV, sodium valproate (300 mg/kg); VitC, vitamin C (50 mg/kg); GABA, gamma amino butyric acid; GABA-T, gamma amino butyric acid transaminase; AchE, acetylcholinesterase; BchE, butyrylcholinesterase.

Oral administration of the *Mimosa pudica* aqueous extract in mice significantly decreased the activity of GABA-transaminase [F (7, 40) = 118.1; P < 0.01] in the hippocampus. The plant extract significantly inhibited this activity by 48.19% and 60.01% at the doses of 80 and 160 mg/kg, respectively. The effects of the *Mimosa pudica* aqueous extract were similar to those of sodium valproate (300 mg/kg; 62.05% inhibition) and vitamin C (50 mg/kg; 57.32% inhibition).

During the early phases of pilocarpine–picrotoxin-induced seizures, cholinergic neurotransmission is further stimulated and excitotoxicity triggered. Oral administration of the *Mimosa pudica* aqueous extract significantly inhibited (p < 0.05) the activity of acetylcholinesterase, which was inhibited by 25.77% and 25.44% at the doses of 80 and 160 mg/kg, respectively (Table 3). This inhibitory action was similar to that of sodium valproate (300 mg/kg; 22.42% inhibition) and vitamin C (50 mg/kg; 27.39% inhibition) (Table 3). The plant extract significantly inhibited (p < 0.05) the activity of butyrylcholinesterase by 33.67% and 31.96% at the doses of 80 and 160 mg/kg, respectively. This inhibitory activity was similar to that of 300 mg/kg sodium valproate (32.03% inhibition) or 50 mg/kg vitamin C (37.23% inhibition) (Table 3).

#### 3.5.2 Effects of the *Mimosa pudica* aqueous extract on the levels of MDA, GSH, and NO and the activities of CAT and SOD

As shown in Table 4, the levels of MDA in the brain were strongly increased in the negative control group of mice compared to the normal group. *Mimosa pudica* induced a sustained and dose-related antioxidant effect by inhibiting the MDA production [F (7, 40) = 81.71; P < 0.001] (Table 4). The calculated results revealed that the brain MDA level in the negative control group was 0.48 ± 0.01 μmol/g. The brain MDA levels were significantly decreased to 0.24 ± 0.01 μmol/g (P < 0.05) and 0.18 ± 0.03 μmol/g, (P < 0.01), respectively, for 80 and 160 mg/kg extract-treated groups. Similarly, sodium valproate at 300 mg/kg decreased MDA levels from 0.48 ± 0.01 μmol/g in the negative control group to 0.19 ± 0.01 μmol/g same as with vitamin C at 50 mg/kg, where it decreased from 0.48 ± 0.01 to 0.16 ± 0.01 μmol/g, (P < 0.01) (Table 4).

**TABLE 4:** Effects of the *Mimosa pudica* aqueous extract on the concentrations of MDA, GSH, and NO and the activities of CAT and SOD of pilocarpine–picrotoxin-treated mice.

Parameter	NoG	NCG	*Mimosa pudica* (mg/kg)				SoV (mg/kg)	VitC (mg/kg)
			20	40	80	160	300	50
MDA (μmol/g)	0.18 ± 0.02^b^	0.48 ± 0.01	0.34 ± 0.02	0.27 ± 0.02^a^	0.24 ± 0.01^a^	0.18 ± 0.03^b^	0.19 ± 0.01^b^	0.16 ± 0.01^b^
NO (μmol/mg protein)	1.28 ± 0.36^b^	2.97 ± 0.12	2.73 ± 0.47	2.48 ± 0.42	1.50 ± 0.14^a^	1.26 ± 0.36^b^	1.25 ± 0.42^b^	1.21 ± 0.33^b^
GSH (μmol/mg protein)	7.86 ± 0.46^c^	2.72 ± 0.62	3.86 ± 0.58^a^	4.33 ± 0.78^b^	7.04 ± 1.23^c^	7.99 ± 0.65^c^	8.22 ± 1.17^c^	8.03 ± 0.69^c^
SOD (U/mg protein)	16.71 ± 1.49^a^	12.95 ± 0.97	13.36 ± 1.64	14.79 ± 0.77	15.82 ± 1.35^a^	15.82 ± 1.20^a^	15.26 ± 1.43^a^	17.64 ± 2.00^a^
CAT (U/mg protein)	0.28 ± 0.06	0.17 ± 0.03	0.27 ± 0.00	0.26 ± 0.03	0.30 ± 0.05^a^	0.31 ± 0.03^a^	0.30 ± 0.06^a^	0.31 ± 0.06^a^

Results are expressed as mean ± S.E.M. n = 6 animals. Statistical differences were tested using one-way ANOVA, followed by Tukey’s (HSD) multiple comparison test. Significant difference set at ^a^P < 0.05, ^b^P < 0.01, and ^c^P < 0.001 versus the negative control group. NCG, negative control group received distilled water. NoG, normal group received distilled water; SoV, sodium valproate; VitC, vitamin C; MDA, malondialdehyde; NO, nitric oxide; SOD, superoxide dismutase; GSH, glutathione; CAT, catalase. All mice were subjected to epileptogenesis induced by pilocarpine (360 mg/kg) except the normal group. *M. pudica*, *Mimosa pudica*.

As shown in Table 4, the brain of both the positive control group of mice and animals treated with the *Mimosa pudica* aqueous extract presented a constant decrease in NO activity [F (7, 40) = 16.09; P < 0.001]. The brain of the animal receiving the *Mimosa pudica* aqueous extract at doses of 80 mg/kg (1.50 ± 0.14) and 160 mg/kg (1.26 ± 0.36) μmol/mg protein (P < 0.01) showed a significant decrease in the NO concentration at the end of the test when compared to control mice, which is 2.97 ± 0.12 μmol/mg protein. Similarly, sodium valproate and vitamin C administered at doses of 300 mg/kg and 50 mg/kg decreased the brain NO activity from 2.97 ± 0.12 μmol/mg protein in the negative control group to 1.25 ± 0.42 and 1.21 ± 0.33 μmol/mg protein (P < 0.01), respectively.

Table 4 presents the level of brain GSH. The brain GSH was significantly lower (p < 0.001) in the negative control group administered with pilocarpine–picrotoxin (2.80 ± 0.62 *μ*mol/mg protein) when compared to that of the normal group (7.87 ± 0.47 *μ*mol/mg protein). The estimation of glutathione activity revealed that at doses of 80 and 160 mg/kg, the *Mimosa pudica* aqueous extract significantly optimised the production of brain GSH to 7.04 ± 1.23 and 7.99 ± 0.65 μmol/mg protein (P < 0.001), respectively, in comparison with the low production of 2.72 ± 0.62 μmol/mg protein in the parasitized treated group of mice. Likewise, sodium valproate at a dose of 300 mg/kg alone also caused a significant increase of 8.22 ± 1.17 μmol/mg protein (P < 0.001), similar to that of vitamin C of 8.03 ± 0.69 μmol/mg protein (P < 0.001).

The report of the SOD activity shows that the aqueous extract *Mimosa pudica* acted as a spontaneous stimulant of antioxidant activity [F (7, 40) = 4.49; P < 0.01]. Pre-treatment of mice with graded doses of the aqueous extracts significantly triggered an increase in SOD activity in 80 mg/kg (15.82 ± 1.35) and 160 mg/kg (15.82 ± 1.20) U/mg protein, (P < 0.05) in opposition to the control animals, where the production was instead retarded with a maximum at 12.95 ± 0.97. In accordance with the extract-treated groups, sodium valproate at 300 mg/kg alone and vitamin C also triggered a significant increase to 15.26 ± 1.43 and 17.64 ± 2.00 U/mg protein (P < 0.05), respectively (Table 4).

A significant augmentation in the brain CAT activity [F (7, 40) = 4,08; P < 0.01] in the animals treated with the aqueous extract of *Mimosa pudica* (80 mg/kg, p.o.) [0.30 ± 0.05 U/mg protein, P < 0.05], (160 mg/kg, p.o.) [0.31 ± 0.03 U/mg protein, P < 0.05], vitamin C (50 mg/kg, p.o.) [0.30 ± 0.06 U/mg protein, P < 0.05], and sodium valproate (300 mg/kg, i.p.) [0.31 ± 0.06 U/mg protein, P < 0.05] was observed 1 h after oral administration (Table 4).

#### 3.5.3 Effects of the *Mimosa pudica* aqueous extract on the brain-derived neuro factor

**FIGURE 5:**
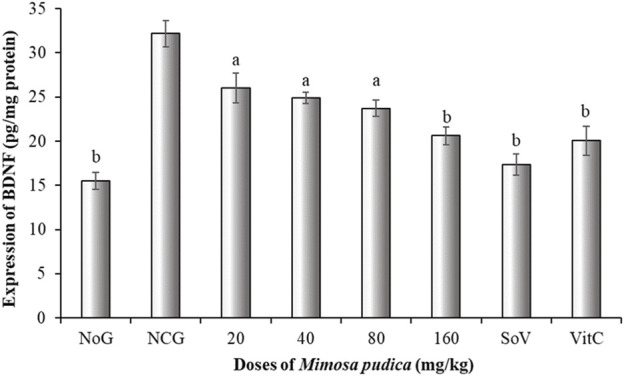
Effects of the *Mimosa pudica* aqueous extract on brain-derived neuro factor expression of pilocarpine–picrotoxin-treated mice. Results are expressed as mean ± S.E.M. n = 6 animals. Statistical differences were tested by a one-way ANOVA, followed by Tukey’s (HSD) multiple comparison test; the differences were considered significant at ^a^P < 0.05 and ^b^P < 0.01, versus the negative control group. NoG, normal group treated with distilled water; NCG, negative control group treated with distilled water; SoV, sodium valproate (300 mg/kg); VitC: vitamin C (50 mg/kg). All mice were subjected to epileptogenesis induced by pilocarpine (360 mg/kg) except the normal group.

The negative control group of mice expressed an increment of BDNF expression. Surprisingly, administration of *Mimosa pudica* extracts exerted a significant inhibitory effect by slowing BDNF production in the brain [F (7, 40) = 67.54; P < 0.001] (Figure 5). The estimation of BDNF levels revealed that at doses of 80 and 160 mg/kg, the *Mimosa pudica* aqueous extract significantly slowed BDNF production to 23.72 ± 0.90 and 20.61 ± 1.01 pg/mg protein, respectively, in comparison to the high-production 32.17 ± 1.47 pg/mg protein in the negative control group of mice. Likewise, sodium valproate at 300 mg/kg alone also induced a significant reduction of this concentration to 17.33 ± 1.18 pg/mg protein (P < 0.01), as well as vitamin C, where the concentration was 20.05 ± 1.66 pg/mg protein (P < 0.01) (Figure 5).

### 3.6 Acute oral toxicity study of the *Mimosa pudica* aqueous extract in naive mice

The *Mimosa pudica* aqueous extract was administered to two groups of mice (female and male). Mice in each group were carefully examined for any signs of toxicity (behavioural changes and mortality) for 14 days (Table 5). There were no deaths or any signs of toxicity observed after oral administration of single doses of extract at any dose level up to the highest dose tested (5,000 mg/kg). This did not allow us to determine LD_50_.

**TABLE 5:** Acute toxicity of the *Mimosa pudica* aqueous extract administered orally to different groups of female and male mice.

Treatment	Dose (mg/kg)	Toxicity signs	Mortality latency (h)	D/T mice	
				Male	Female
Distilled water		None	—	0/3	0/3
*Mimosa pudica*	500	None	—	0/3	0/3
	1,000	None	—	0/3	0/3
	2,000	None	—	0/3	0/3
	5,000	None	—	0/3	0/3

D/T, dead/treated mice; None, no toxic symptoms during the observation period; mortality latency, time to death after the oral administration. Control group received distilled water (10 mL/kg, *per os*). D/T, death/treated mice; *M. pudica*, *Mimosa pudica*.

## 4 Discussion

Epilepsy strongly alters the living condition of individuals worldwide and particularly in the emerging state (Tang et al., 2017). Epilepsy was induced artificially in the laboratory by intraperitoneal injection with pilocarpine in mice at 360 mg/kg, a dose that triggers seizures. The derived metabolites existing in natural products are greatly valorised for their prophylactic and therapeutic actions against epilepsy. This study was elaborated to assess the anticonvulsant and antiepileptogenic properties of an aqueous extract of *Mimosa pudica* in mouse model epilepsy: oxidative stress metabolism pathways, cholinergic, GABAergic, and BDNF signalling. The outcome of the anticonvulsant and antiepileptogenic test performed on mice pre-treated orally with the *Mimosa pudica* aqueous extract (80 and 160 mg/kg) and the intraperitoneal injection with pilocarpine–picrotoxin depicted that the extract did not alter the occurrence of seizures; nevertheless, the extract considerably attenuated seizure development in mice through the increment of latency to the *status epilepticus,* 60 min post-treatment. Small quantities of picrotoxin were administered to mice 24 h after pilocarpine injection to reactivate pilocarpine and reinforce convulsion. Once again, the treatment raised the time of onset of first clonic seizures and generalized clonic–tonic seizures, as well as the decrement of the number and duration of clonic seizures and generalized tonic–clonic seizure. Moreover, the extract significantly increased the score of generalized tonic–clonic seizures. This effect is analogous to that of sodium valproate, the typical antiepileptic remedy. Sodium valproate is capable to raise the brain GABA content via the obstruction of GABA reuptake, the blockage of GABA transaminase, and the stimulation of GABA decarboxylase (Davis et al., 2000; Hoffmann et al., 2008). Medication that suppresses pilocarpine–picrotoxin-induced convulsions usually relieve temporal lobe epilepsy (Arshad and Naegele, 2020). It can be suggested that the antiepileptogenic and anticonvulsant properties of the plant extract could be mediated by the GABA receptor complex and by enhancing GABA receptor neurotransmission (Greenfield, 2013; Silverman, 2018). Furthermore, the presence of anticonvulsant metabolites such as flavonoids in the extract could explain this effect. With respect to neurological disorders, including infantile convulsions, temporal lobe epilepsy, generalised tonic–clonic epilepsy, many flavonoids are known to provide anticonvulsant and antiepileptogenic activities (Kwon et al., 2019). Emerging evidence suggests that the beneficial effects of flavonoids on epileptic seizures may be associated with modulation of the GABA complex receptors (Hanrahan et al., 2015; Wasowski and Marder, 2012).

The open-field test displayed that the aqueous extract triggered an intensification of crossing, grooming, and the motionless interval at the middle of the open field and declined the number of rearing and faecal boli defecated, establishing an upgrading locomotory and exploratory behaviour in mice improving the locomotor activity (Augustsson, 2004; Bum et al., 2009; Moto et al., 2013).

The rotarod test was conducted in mice, and the results showed that the administration of the *Mimosa pudica* aqueous extract (80–160 mg/kg) impressively prolonged the period of immobilization on the rotarod bar and diminished the recurrences of falls. This behavioural adaptation reveals the capacity of the plant extract to prevent animals from any motor dysfunction (Botton et al., 2010).

The outcome of the catalepsy test revealed that the latency to the initiation of voluntary movement was greatly enhanced, extending the duration of akinesia and other major tranquilising processes. These observations corroborated that the daily administration of the *Mimosa pudica* aqueous extract induced a sedative effect, as observed in the incapacity and inability of the mice to initiate motor action (Taiwe et al., 2016a).

The major hostile effect of pilocarpine-induced seizures is the decline of GABA levels and proliferation of GABA-transaminase activity (Jobin and Paulose, 2010). Epilepsy propagation is shut down by GABA, an inhibitory neurotransmitter used as an index of physiological and pathological statuses in the brain. However, this inhibitory effect of GABA is antagonised by GABA-transaminase, which hydrolyses GABA in the synapse (Ding et al., 2011; Zhang et al., 2006). Oral administration of the *Mimosa pudica* aqueous extract significantly improved the brain GABA levels and limited the activity of GABA-transaminase, which suggested an anticonvulsant activity of the extracts (Hosking and Roff Hilton, 2002; Mason et al., 2001). This effect of *Mimosa pudica* could be interrelated to the existence of secondary metabolites in the extract stimulating the GABA_A_ receptor complex to alleviate GABA-transaminase activity and activate glutamic acid decarboxylase to synthetise GABA, or inhibition of GABA reuptake, thereby promoting GABAergic neurotransmission in the brain (Hashimoto et al., 2006; Kanaani et al., 2010).

Motor and locomotion incoordination, memory deficit, and exploratory disorders originate from a decrease in the number of brain cells in the hippocampal CA1 region (Matilla et al., 1998). In cholinergic neurotransmission, acetylcholinesterase (AchE) constitutes a neurochemical implicated in decrease retention, and its overload is associated with memory impairment (Bachurin et al., 2017). The AchE mechanism of action consists of enzymatic degradation of acetylcholine (Ach) into acetic acid and choline, which are less active in the memory process and exploratory behaviour, resulting in cognitive deficiency (Blake et al., 2014). In the pilocarpine-induced motor and locomotion incoordination, the acetylcholinesterase and butyrylcholinesterase levels rise considerably. Interestingly, the results obtained from this study indicated that *Mimosa pudica* aqueous extracts inhibited the activities of AchE and BchE. In this regard, we could assume that the aqueous extract possesses a compound that interferes with the cholinergic and noradrenergic systems (Botton et al., 2010; Koutseff, 2011), as well as the serotonergic and glutamatergic systems (Horiguchi et al., 2011). Thus, inhibiting the acetylcholinesterase activity slows the hydrolysis of acetylcholine and increases its availability in the synapse, thereby multiplying its binding on the post-synaptic membrane receptors at the level of the cerebral cortex and the hippocampus, prolonging and amplifying its cholinergic neurotransmission to facilitate learning, memory, and exploratory behaviour observed in the open-field paradigm (Bordet, 2003; Viaules, 2016).

Epilepsy is a disproportionate electrical firing of neurons associated with unstable atom production, leading to oxidative stress (Sejima et al., 1997; Waldbaum and Patel, 2010). Pilocarpine–picrotoxin-induced convulsions boost the oxidative stress biomarker activity such as MDA levels and NO in the hippocampus of mice. Lipid peroxidation results in MDA release, which the overload of this marker serves as a sign of oxidative stress and cell denaturation by degradation of biomolecules such as lipids, proteins, and nucleic acids (Ding et al., 2011; Shin et al., 2011), while a decrease serves as a sign of antioxidant effects of the extract. A study on quercetin demonstrated that it possesses antioxidant effects by slowing ROS formation during oxidation of polyunsaturated fatty acids, thus limiting cell apoptosis and their adverse effects (Suematsu et al., 2011). Our finding disclosed that the *Mimosa pudica* aqueous extract powerfully detoxified MDA by reducing its level in plasma, suggesting that the antioxidant effect of the plant extract could be mediated by the quercetin compounds.

Nitric oxide is a free radical in living organisms used as an index of oxidative stress. Inductors of epilepsy stimulate NMDA receptors to liberation calcium, which, in turn, enhances neuronal NOS expression via the stimulation of calcium–calmodulin pathways. Under the influence of monooxygenase contained in neuronal nitric oxide synthetase (nNOS) and in the absence of retro-control, L-arginine will be oxidised into L-citruline, which, in turn, participates in the formation of nitric oxide (NO) radicals (Förstermann and Sessa, 2012; Meffert, 1997). Pilocarpine strongly boosted the production of NO radicals; pre-treatment of mice with the *Mimosa pudica* aqueous extract drastically slowed the formation of NO radicals. This attenuating action could be an indicator that the extract is partially capable of scavenging NOS or suppressing their formation (Puttachary et al., 2015; Yao et al., 2022).

The cytosolic thiol glutathione (GSH), a non-enzymatic antioxidant, when it gets converted to the oxidized form, catalyses the reduction of hydrogen peroxide and improves its viability and, therefore, prevents the formation of hydroxyl radicals (Lushchak, 2012). The decreased concentration of GSH is an indicator of lipid peroxidation. Our plant extracts significantly accelerate the production of GSH in mice. From this finding, it can be suggested that the antioxidant effects of the *Mimosa pudica* aqueous extract are due to the existence of bioactive compounds that mimic the sodium valproate mechanism of action.

SOD is an enzymatic antioxidant which interacts with free radicals, especially superoxide, by catalysing its conversion into hydrogen peroxide to limit its accumulation, thus preventing their detrimental effect and the damage of cells (Ono et al., 2000; Schulz et al., 2000). The pilocarpine-injected mice showed a drastic decrease in the SOD activity. The outcome of the treatment with the plant extract is the restoration of the initial level of SOD activity in the brain tissues of pilocarpine-injected mice, thus enhancing the protection of cell membranes (Freitas, 2009; Freitas et al., 2005; Wang et al., 2009).

The brain, with its high oxygen consumption and the high level of lipid peroxidation, is extremely susceptible to oxidative stress. Catalase stands out as a paramount enzymatic antioxidant. It effectively catalyses the breakdown of hydrogen peroxide into water and oxygen, a potentially harmful byproduct of cellular metabolism. This reaction detoxifies hydrogen peroxide and prevents oxidative damage (Anwar et al., 2024; Kang et al., 2013). Pilocarpine–picrotoxin-induced epileptic convulsions generated a significant reduction in the catalase activity. Interestingly, the collapse in catalase activity was significantly improved by treatment with the *Mimosa pudica* extract at the doses 80–160 mg/kg, suggesting that the extract may partially contain some compounds that could activate catalase or scavenge superoxide anions, hydroxyl radicals, and hydrogen peroxide.

The fundamental mechanisms of connections in neurons and neuroproliferation are strongly influenced by neurotrophins (Lim et al., 2015). The ascent of BDNF signalling initiated by pilocarpine-induced seizure promotes a compensatory neuroplastic response to neuron damage accompanied with neurodegeneration (Wang et al., 2009). In contrast, treatment of mice with the *Mimosa pudica* aqueous extract significantly decreased BDNF expressions in pilocarpine–picrotoxin-injected mice. These results suggest that the plant extract can suppress the compensatory adaptative response of epileptogenesis (Lim et al., 2015).

In the acute toxicity test, no deaths or any signs of toxicity were observed after the oral administration of a single dose of the *Mimosa pudica* extract at any dose level up to the highest dose tested in mice, thus suggesting that DL_50_ is above 5,000 mg/kg. An extract with a DL_50_ value higher than 5,000 mg/kg *p.o*. is considered non-toxic or relatively non-toxic. This result corroborated with that of Nghonjuyi et al. (2016), who reported that the extract of *Mimosa pudica* had a DL_50_ value higher than 5,000 mg/kg when administered orally.

As a limitation to this work, the effects of the *Mimosa pudica* extract in mouse models of temporal lobe epilepsy induced by pilocarpine–picrotoxin without generalisation of seizures in the hippocampus and cortex but with nonconvulsive focal seizures were not evaluated since the primary aim of our study is to identify only the effects of the *Mimosa pudica* extract aqueous extract on pilocarpine–picrotoxin models of temporal lobe epilepsy, oxidative/nitrosative stress, and alteration in GABAergic/cholinergic pathways and BDNF expression.

## 5 Conclusion

In summary, this study showed that the aqueous extract of *Mimosa pudica* leaves and stems possesses potent antiepileptogenic and anticonvulsant activities against pilocarpine–picrotoxin-induced temporal lobe epilepsy that may be directly related to the increase in protection against *status epilepticus* and generalized tonic–clonic seizures, through the amelioration of GABAergic and cholinergic transmission pathways, attenuation of oxidative/nitrosative stress, and the normalisation of brain-derived neurotrophic factor expression.

## Data Availability

The raw data supporting the conclusions of this article will be made available by the authors, without undue reservation.
